# Preoperative pelvic floor muscle exercise for early continence after holmium laser enucleation of the prostate: a randomized controlled study

**DOI:** 10.1186/s12894-019-0570-5

**Published:** 2020-01-23

**Authors:** Go Anan, Yasuhiro Kaiho, Hiromichi Iwamura, Jun Ito, Yuki Kohada, Jotaro Mikami, Makoto Sato

**Affiliations:** Department of Urology, Tohoku Medical and Pharmaceutical University, Sendai, Japan, 1-15-1 Fukumuro, Miyagino-ku, Sendai, Miyagi 983-8536 Japan

**Keywords:** Benign prostatic hyperplasia, Incontinence, HoLEP, Pelvic floor muscle exercise

## Abstract

**Background:**

Transient postoperative urinary incontinence is a bothersome complication of holmium laser enucleation of the prostate (HoLEP). The effects of preoperative pelvic floor muscle exercise (PFME) for early recovery of continence after HoLEP have never been elucidated. The aim of this study was to determine the benefit of preoperatively started PFME for early recovery of continence after HoLEP.

**Methods:**

We randomly assigned patients to start PFME preoperatively and continue postoperatively (group A) or start PFME no earlier than the postoperative period (group B). The primary outcome was time to complete urinary control, defined as no pad usage. The secondary outcome was measured using the International Consultation on Incontinence Questionnaire-Short Form (ICIQ-SF) score. Univariate and multivariate analyses were performed to identify parameters associated with recovery of continence after HoLEP.

**Results:**

Seventy patients were randomized across groups A (*n* = 35) and B (*n* = 35). Patients’ characteristics were not different between groups A and B. The postoperative urinary incontinence rate significantly decreased in group A compared with that in group B at 3 months postoperatively [3% vs. 26% (*P* = 0.01)]. However, there were no significant differences between groups A and B at 3 days [40% vs. 54% (*P* = 0.34)], 1 month [37% vs. 51% (*P* = 0.34)], and 6 months [0% vs. 3% (*P* = 1.00)] postoperatively, respectively. The postoperative ICIQ-SF score was not significantly different between groups A and B at any time point postoperatively. In univariate analysis, patients who performed preoperative PFME had a 0.56-fold lower risk of urinary incontinence 1 month after HoLEP and a 0.08-fold lower risk of urinary incontinence 3 months after HoLEP.

**Conclusions:**

Preoperatively started PFME appears to facilitate improvement of early urinary continence after HoLEP.

**Trial registration:**

The study was registered with the University Hospital Medical Information Network Clinical Trials Registry in Japan (UMIN000034713); registration date: 31 October 2018. Retrospectively registered.

## Background

Benign Prostatic Hyperplasia (BPH) is a common condition in elderly males and affects over half of all men aged > 60 years [1]. Holmium laser enucleation of the prostate (HoLEP) is widely used as a minimally invasive surgical procedure because it can be performed in patients with large-sized prostatic hyperplasia and is associated with lower volumetric blood loss and shorter periods of indwelling catheterization and hospitalization than transurethral resection of the prostate (TURP) [2]. However, postoperative transient urinary incontinence has been reported as a bothersome complication of HoLEP in 16–44% patients within 3 months [3–5]. Postoperative urinary incontinence remains a representative complication of HoLEP and can have a negative influence on the patient’s quality of life (QOL) [5].

Pelvic floor muscle exercise (PFME) is one of the physiotherapies used for recovery of continence after prostate surgery. Although the efficacy of PFME for urinary incontinence after prostate surgery remains controversial [6–11], some recent studies demonstrated that PFME started preoperatively and continued postoperatively was associated with better outcomes than PFME started only postoperatively [6–8]. We herein present the first randomized clinical trial to compare the effects of preoperatively started PFME with postoperatively started PFME for early recovery of continence after HoLEP.

## Methods

### Study design

This randomized, prospective study enrolled patients with BPH who underwent HoLEP at a single institution (Tohoku Medical and Pharmaceutical University Hospital) between September 2017 and March 2019. Patients were randomly assigned in a ratio of 1:1 to one of the two groups using a simple randomization procedure (computerized random numbers generated using Microsoft Excel for Windows). Group A included patients who started PFME preoperatively 28 days before HoLEP and continued postoperatively. Group B included patients who started PFME postoperatively only.

The inclusion criteria were males between 50 and 90 years of age with symptoms of dysuria for ≥3 months before study entry. Patients with prostate volumes of ≥30 ml were eligible. Patients who could continue PFME on their own were eligible. We excluded patients who could not continue PFME on their own and those who had severe incontinence before HoLEP due to severe cerebrovascular disorder or spinal cord injuries.

The primary outcome measure was self-reported continence postoperatively. The condition of no incontinence was evaluated by defining complete urinary control as no pad usage. The secondary outcome measure gauged QOL as determined by International Consultation on Incontinence Questionnaire-Short Form (ICIQ-SF) score [12].

Urinary incontinence was evaluated on the day before surgery; 3 days after HoLEP, which was one day after catheter removal; and 1, 3, and 6 months after HoLEP. ICIQ-SF score was evaluated on the day before surgery and at 1, 3, and 6 months after HoLEP. Patients in group A received sufficient instructions for PFME by urological nurses to start the same 28 days before HoLEP and continue thereafter. Then, on the second day after HoLEP, which was the day of catheter removal, all patients in both groups A and B were instructed regarding PFME by the nurses sufficiently. For all patients, the instructions for PFME included illustrations; all patients were instructed to perform a set of 3 min at least three times a day and record it in a PFME performance table. The table was described by all patients, and the PFME implementation status was confirmed for each outpatient.

The ethical committee of Tohoku Medical and Pharmaceutical University Hospital School of Medicine, Sendai, Japan approved the study protocol. Written informed consent was obtained from all patients prior to participation in this study. This study was registered with the Tohoku Medical and Pharmaceutical University Hospital Medical Research Registry in Japan (Protocol 2017–2-056) on August 22, 2017 and was registered with the University Hospital Medical Information Network Clinical Trials Registry in Japan (UMIN000034713) on October 31, 2018.

### Surgical technique

The enucleation procedure was performed following the anteroposterior dissection HoLEP method (antegrade separation method), as reported by Endo et al. [13]. The three lobes technique was used in all cases. In this study, all surgeons used the same surgical techniques. We removed the urinary catheter on the second day after HoLEP and confirmed self-urination and degree of urinary incontinence.

### Predictive factors for postoperative urinary incontinence

Uni- and multivariate analyses were performed to investigate the predictive factors for postoperative urinary incontinence at 3 days and 1, 3, and 6 months after HoLEP, including potential factors. These factors included age, body mass index (BMI), preoperative international prostate symptom score (IPSS) and overactive bladder symptom score (OABSS), ICIQ-SF score, prostate volume, operation time, enucleate prostate weight, presence or absence of diabetes mellitus, and G8 score [14]. Urodynamic examination, including free uroflowmetry, filling cystometry, and pressure flow studies, was performed before HoLEP. We investigated the maximum detrusor pressure, detrusor overactivity, volume at the first desire to void, maximum cystometric capacity, and residual volume.

### Statistical analysis

Prior to this study, there were about 40% postoperative urinary incontinence 1–3 months after HoLEP at our institute. Therefore, between the study groups, we set a significant difference in the urinary incontinence rate of 16–20% as the threshold for clinical importance. This threshold was derived following discussions between clinicians and the project management group as well as inspection of the urinary incontinence rate reported in previous studies that reviewed the number of men who showed urinary incontinence at 1–3 months after HoLEP [3–5]. Using the two-sided test to differentiate between proportions, this study had an ability of 80% to detect a difference of 20% in the proportion of patients remaining incontinent at 1 and 3 months after HoLEP, assuming a total sample size of 70 patients and a type 1 error rate of 0.05.

Patient characteristics are described in terms of mean and standard deviation (SD) or range for continuous variables. We used the JMP version 9.0 (SAS Institute Inc., Cary, NC. USA) for statistical analyses. Intergroup differences were analyzed using Mann–Whitney U test for continuous variables and Fisher exact tests for categorical variables. Multivariate analysis was analyzed using logistic regression analysis. *P* value of < 0.05 was considered statistically significant.

## Results

**Fig. 1:**
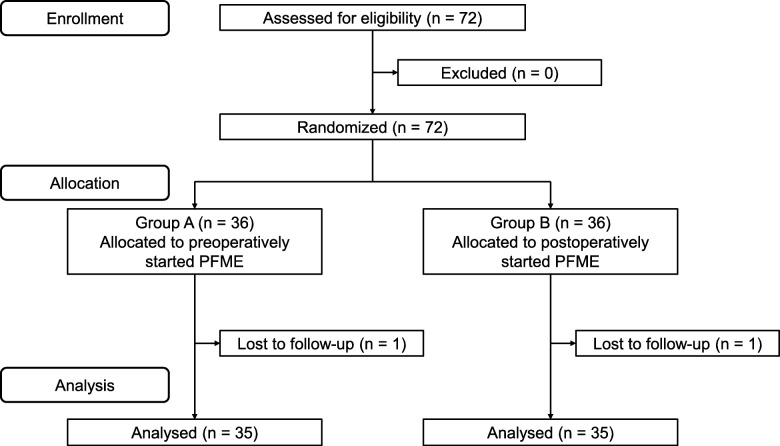
The study flow diagram. PFME: pelvic floor muscle exercise

**Fig. 2:**
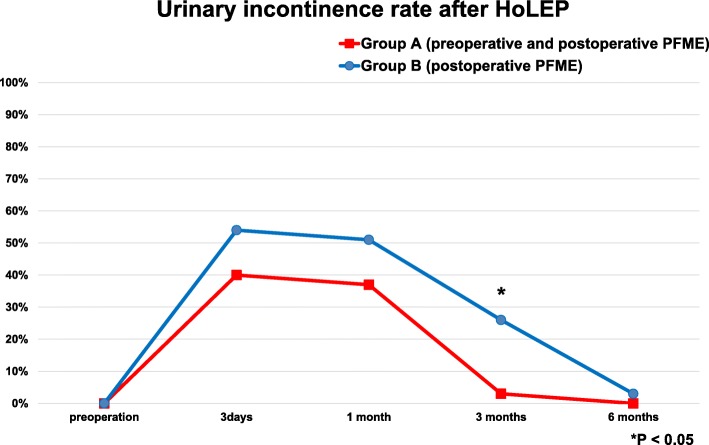
Comparison of postoperative urinary incontinence rate after HoLEP between group A (preoperative and postoperative PFME) and group B (postoperative PFME alone). PFME: pelvic floor muscle exercise

A total of 70 patients were randomized into groups A (PFME started preoperatively and continued thereafter, *n* = 35) and B (PFME postoperatively only, *n* = 35) for final analysis (Fig. 1). Among the patient and perioperative background characteristics, there were no differences in age, BMI, IPSS, OABSS, ICIQ-SF score, prostate volume, operation time, enucleate prostate weight, diabetes mellitus status, and G8 score between the two groups (Table 1). The postoperative urinary incontinence rate was significantly lower in group A than in group B at 3 months postoperatively (3% vs. 26%; *P* = 0.01); however, there were no significant differences in the urinary incontinence rate between groups A and B at 3 days (40% vs. 54%; *P* = 0.34), 1 month (37% vs. 51%; *P* = 0.34), and 6 months (0% vs. 3%; *P* = 1.00) postoperatively (Fig. 2). Furthermore, the postoperative ICIQ-SF score was not significantly different between groups A and B at 1 month (5.4 ± 4.9 vs. 5.6 ± 4.9; *P* = 0.89), 3 months (2.9 ± 3.4 vs. 3.8 ± 4.6; *P* = 0.80), and 6 months (1.5 ± 2.0 vs. 1.5 ± 2.4; *P* = 0.83) postoperatively.

**Table 1:** Patients background (median, range)

	Group A	Group B	*P*-value
Number of cases	35	35	
Age (years)	72 (62–83)	73 (57–86)	0.55
BMI (kg/m^2^)	24 (17–31)	24 (19–32)	0.37
G8	15 (12–16)	14 (12–17)	0.60
IPSS	17 (5–34)	19 (5–35)	0.32
OABSS	6 (2–13)	6 (2–15)	0.94
ICIQ-SF score	0 (0–13)	0 (0–13)	0.83
Diabetes mellitus (n, %)	12 (34%)	10 (29%)	0.80
Prostate volume (mL)	56 (35–208)	60 (35–114)	0.89
Operation time (min)	89 (50–202)	88 (34–160)	0.88
Enucleate prostate weight (g)	31 (7–168)	34 (5–94)	0.81

*BMI* Body mass index, *ICIQ-SF* International consultation on incontinence questionnaire-short form, *IPSS* International prostate symptom score, *OABSS* Overactive bladder symptom score

We investigated the predictive factors for urinary incontinence at 3 days and 1, 3, and 6 months after HoLEP. As shown in Table 2, by univariate and multivariate analyses, preoperative PFME was a significant predictive factor for urinary incontinence at 3 months after HoLEP [odds ratio (OR), 0.08; *P* = 0.01 (univariate), OR, 0.10; *P* = 0.01(multivariate)]. At 3 days and 1 month after HoLEP, there were no significant predictive factors for urinary incontinence. At 6 months postoperatively, predictive factors could not be evaluated and were thus not included in Table 2 because there remained only one case with urinary incontinence in 70 cases. In univariate analysis, patients who performed preoperative PFME had a 0.56-fold lower risk of being incontinent 1 month after HoLEP and a 0.08-fold lower risk of being incontinent 3 months after HoLEP (Table 2).

**Table 2:** Predictive factors for urinary incontinence at 3 days, 1 month, and 3 months after HoLEP

Variable			Univariate			Multivariate	
		Odds ratio	95%CI	*P*-value	Odds ratio	95%CI	*P*-value
	3 days	0.82	0.31-2.20	0.80	0.83	0.30-2.28	0.72
Age (years) (≧75 vs <75)	1 month	1.26	0.47-3.37	0.80	1.34	0.48-3.76	0.57
	3months	2.00	0.52-7.72	0.48	1.42	0.29-6.56	0.65
	3 days	1.24	0.48-3.17	0.81			
BMI (kg/m^2^) (≧24 vs <24)	1 month	0.76	0.29-1.97	0.63			
	3months	1.96	0.50-7.68	0.50			
	3 days	0.53	0.19-1.48	0.30			
Diabetes mellitus (yes vs no)	1 month	1.07	0.39-2.96	1.00			
	3months	1.56	0.39-6.19	0.71			
	3 days	0.83	0.32-2.15	0.81	0.80	0.30-2.10	0.65
Prostate volume (mL) (≧60 vs <60)	1 month	1.67	0.64-4.35	0.34	1.66	0.63-4.46	0.30
	3months	2.44	0.56-10.7	0.29	2.43	0.54-13.2	0.25
	3 days	0.78	0.30-2.00	0.64			
Operation time (min) (≧90 vs <90)	1 month	1.21	0.47-3.13	0.81			
	3months	3.27	0.77-13.9	0.17			
	3 days	1.01	0.35-2.91	1.00			
Enucleate prostate weight (g) (≧50 vs <50)	1 month	2.13	0.73-6.21	0.19			
	3months	3.29	0.83-13.0	0.12			
	3 days	0.56	0.22-1.45	0.34	0.51	0.19-1.33	0.17
Pelvic floor muscle exercise (preoperative vs postoperative)	1 month	0.56	0.22-1.45	0.34	0.54	0.20-1.42	0.21
	3months	0.08	0.01-0.71	0.01	0.10	0.005-0.60	0.01

*BMI* Body mass index, *HoLEP* Holmium laser enucleation of the prostate

Regarding the urodynamic examination results, there were no differences between groups A and B in terms of the maximum detrusor pressure (76 ± 33 cmH_2_O vs. 73 ± 29 cmH_2_O; *P* = 0.85), detrusor overactivity (18% vs. 12%; *P* = 0.73), volume at the first desire to void (274 ± 118 ml vs. 288 ± 145 ml; *P* = 0.79), maximum cystometric capacity (422 ± 171 ml vs. 366 ± 180 ml; *P* = 0.12), and residual volume (127 ± 145 ml vs. 135 ± 178 ml; *P* = 0.91). We also investigated the predictive factors for postoperative urinary incontinence among the factors obtained on urodynamic examination, and no significant predictive factors were found at 3 days, 1 month, 3 months, and 6 months after HoLEP.

## Discussion

Urinary incontinence is one of the common complications after prostate surgeries such as radical prostatectomy (RP) for prostate cancer and TURP as well as HoLEP for BPH. Urinary incontinence after surgery is a challenging complication that may discourage patients from seeking surgery and reduces patient QOL [5]. PFME, which was shown to be effective primarily in females with stress urinary incontinence [15], is one of the physiotherapy approaches used for recovery of continence after prostate surgery. However, to the best of our knowledge, no study has examined the effects of PFME on urinary continence after HoLEP. Although the period is relatively short in most patients, postoperative urinary incontinence seriously decreases the postoperative QOL not only after HoLEP but also other prostate surgeries.

The current study demonstrated that preoperatively started PFME promoted early recovery of continence after HoLEP. We started PFME preoperatively to promote early recovery of continence after HoLEP based on several recent studies demonstrating that preoperatively started PFME was associated with improved outcomes compared with postoperatively started PFME in patients undergoing RP [6–8]. A recent meta-analysis showed that preoperatively started PFME significantly reduced the risk of postoperative urinary incontinence by 36% at 3 months after RP [8]. Conversely, the same meta-analysis also showed that there were no differences in long-term outcomes beyond the first 6 months postoperatively, suggesting that preoperatively started PFME might aid in early recovery of short-term continence and not long-term continence [8]. These results regarding preoperatively started PFME in RP are consistent with our findings which show that preoperatively started PFME promoted early recovery of continence at 3 months after HoLEP but did not have an effect on long-term continence.

The mechanism underlying the effect of preoperatively started PFME on urinary incontinence after HoLEP is unclear. One potential explanation is that PFME requires a certain time period to exhibit the beneficial effect. One study previously reported that a minimum of 1 month was required for the increase in the contraction strength of the pelvic floor muscle after PFME [9]. We considered that PFME affected postoperative urinary incontinence after at least 1 month from the start of PFME. Therefore, in the group that underwent preoperative PFME from 1 month before HoLEP, a significant effect on postoperative urinary incontinence was observed 1–3 months after HoLEP. In this study, only at 3 months after HoLEP, there was a significant difference in the urinary incontinence rate between the two groups. The same rationale might also underlie the disappearance of the beneficial effect of PFME by 6 months postoperatively. However, the study sample size was small; therefore, further randomized studies involving larger groups of patients are necessary. Another possibility underlying the benefit of preoperatively started PFME for postoperative urinary incontinence is the additional instruction timing  provided to the patients by the nurses, which might have allowed for improved acquisition of appropriate PFME techniques. Therefore, it is critical for the patients to learn to perform PFME appropriately [16], as noted by several reports emphasizing the importance of repeated education [16, 17].

Several studies examined the effect of PFME on postoperative urinary incontinence in TURP, a standard surgery for BPH, as well as HoLEP. The efficacy of PFME for urinary incontinence after TURP continues to be a topic of debate [9–11]. Chang et al. reported that patients performed postoperative PFME showed improvement in urinary continence at 3 and 4 weeks after TURP compared with patients who did not receive the intervention [9]. Conversely, Glazener et al. reported that PFME started 6 weeks after TURP did not lead to an improvement in urinary continence at 3, 6, 9, or 12 months after TURP [10]. One study investigating preoperatively started PFME found that preoperatively started PFME was not associated with an improvement in urinary continence after TURP compared with the control group [11], which is not consistent with the finding of the current study. Although the initiation time and duration of preoperative PFME were not described in that report, one potential reason for the discrepant finding is short or insufficient exercise duration for the emergence of the beneficial effect of preoperatively started PFME. Another possibility is that patients might have recovered from postoperative urinary incontinence after TURP relatively early; the number of patients with postoperative urinary incontinence in that case would be too low to detect any significant differences regardless of preoperative PFME.

There are several limitations to this study. First, the number of cases was small. Second, we did not investigate the severity, type, or continuation of incontinence using objective tests, such as the pad test. However, we believe that preoperative PFME contributed to the improvement of early urinary continence after HoLEP because the current study was conducted as a prospective randomized trial including two groups with no differences in patient background characteristics. Additionally, the multivariate analysis showed that preoperative PFME was the only significant predictive factor for early recovery of continence at 3 months after HoLEP (Table 2). Nonetheless, larger randomized prospective studies that investigate the efficacy of preoperatively started PFME will be beneficial to further elucidate definitive treatment strategies of PFME for recovery of continence after HoLEP.

## Conclusions

Preoperatively started PFME may promote early recovery of continence at 3 months after HoLEP. Preoperative PFME is a costless and minimally invasive treatment that can be adapted to individuals attempting to recover urinary continence. However, the study sample size was small; therefore, further randomized studies involving larger groups of patients are necessary.

## Data Availability

All the data supporting our findings is contained within the manuscript, any missing details will be shared upon request.

## Abbreviations

BMI: Body mass index
BPH: Benign prostatic hyperplasia
HoLEP: Holmium laser enucleation of the prostate
ICIQ-SF: International Consultation on Incontinence Questionnaire-Short Form
IPSS: International prostate symptom score
OABSS: Overactive bladder symptom score
PFME: Pelvic floor muscle exercise
QOL: Quality of life
RP: Radical prostatectomy
TURP: Transurethral resection of the prostate
